# Multiple overlapping risk factors for childhood wheeze among children in Benin

**DOI:** 10.1186/s40001-022-00919-1

**Published:** 2022-12-26

**Authors:** Euripide F. G. A. Avokpaho, Laure Gineau, Audrey Sabbagh, Eloic Atindégla, Arnauld Fiogbé, Sean Galagan, Moudachirou Ibikounlé, Achille Massougbodji, Judd L. Walson, Adrian J. F. Luty, André Garcia

**Affiliations:** 1 Institut de Recherche Clinique du Bénin, Abomey-Calavi, Benin; 2 ED 393 Pierre Louis de Santé Publique, Université Paris Cité, Paris, France; 3 MERIT, IRD, Université Paris Cité, Paris, France; 4 Ministère de la Santé, Centre National Hospitalo-Universitaire de Pneumo- Phtisiologie, Cotonou, Bénin; 5 DeWorm3, University of Washington, Seattle, WA USA; 6 Department of Global Health, University of Washington, Seattle, WA USA; 7 Centre de Recherche Pour La Lutte Contre Les Maladies Infectieuses Tropicales (CReMIT/TIDRC), Université d’Abomey-Calavi, Abomey-Calavi, Bénin

**Keywords:** Wheeze, Asthma, Air pollution, Open cookstoves, Ascaris infection

## Abstract

**Background:**

The African continent is currently facing an epidemiological transition characterized by a shift from communicable to non-communicable diseases. Prominent amongst the latter are allergies and asthma. In that context, wheeze has multiple potential contributory factors that could include some of the endemic helminth infections, as well as environmental exposures, such as household air pollution. We sought to determine the relative importance of these risk factors among children in Benin.

**Methods:**

We included 964 children aged 6–14 years living in the commune of Comé, south–west Benin. All children were participants in the longitudinal monitoring cohort of the DeWorm3 trial designed to evaluate multiple rounds of community mass treatment with albendazole for interruption of the transmission of soil transmitted helminths (STH). We administered a standard ISAAC questionnaire to determine the presence of wheeze. In addition, we assessed exposure to household air pollution and to other potential allergy-inducing factors, dietary intake and anthropometry. Using STH infection status assessed at the pretreatment baseline timepoint, we used multivariate statistical modelling, controlling for covariates, to investigate associations between wheeze and the different factors measured.

**Results:**

The prevalence of wheezing history was 5.2%, of current wheezing was 4.6% and of severe wheezing was 3.1%, while STH infections were found in 5.6% of children. These profiles did not vary as a function of either age or gender. Infection with *Ascaris lumbricoides,* but not hookworm species, was significantly associated with both current wheeze (adjusted Odds Ratio (aOR) = 4.3; 95% CI [1.5–12.0]) and severe wheeze (aOR = 9.2; 95% CI [3.1–27.8]). Significant positive associations with current wheeze, independent of each other and of STH infection status, were also found for (i) use of open cookstoves (aOR = 3.9; 95% CI [1.3–11.5]), (ii) use of palm cakes for fire lighting (aOR = 3.4; 95% CI [1.1–9.9]), (iii) contact with domestic animals and/or rodents (aOR = 2.5; 95% CI [1.1–6.0]), (iv) being overweight (aOR = 9.7; 95% CI [1.7–55.9]). Use of open cookstoves and being overweight were also independent risk factors for severe wheeze (aOR = 3.9; 95% CI [1.1–13.7]) and aOR = 10.3; 95% CI [1.8–60.0], respectively).

**Conclusions:**

Children infected with *A. lumbricoides* appear to be at elevated risk of wheeze. Deworming may be an important intervention to reduce these symptoms. Improving cooking methods to reduce household air pollution, modifying dietary habits to avoid overweight, and keeping animals out of the house are all additional measures that could also contribute to reducing childrens’ risk of wheeze. Policymakers in LMIC should consider tailoring public health measures to reflect the importance of these different risk factors.

**Supplementary Information:**

The online version contains supplementary material available at 10.1186/s40001-022-00919-1.

## Introduction

Asthma is a chronic lung disease characterized by reversible airway obstruction resulting from an allergic reaction or hypersensitivity resulting in breathing difficulty. The symptoms include wheeze, shortness of breath, chest tightness and cough that vary over time and in intensity [1]. All age groups are affected, but children bear the greatest burden [2]. Asthma is among the top 10 causes of chronic conditions in the global ranking of disability-adjusted life years in participants 5–14 years [3].

In sub-Saharan Africa, infectious diseases continue to be a major driver of overall disease burden in children, although the region is undergoing both an environmental and an epidemiological transition toward an increase in non-communicable diseases (NCD), including increases in allergic diseases and asthma [4–6]. In 2016, air pollution was the second largest risk factor causing NCDs globally, just below tobacco smoking [7], and children aged 5–15 years were three times more likely to die from the combined effects of ambient and household air pollution in Africa’s low- and middle-income countries (LMIC) than globally (12.9 versus 4.1 per 100,000) [8]. Air pollution was responsible for 1.1 million deaths across Africa in 2019, representing 16.3% of all deaths on the continent. The link between air pollution and asthma is complex. Studies have shown that air pollution may induce or aggravate asthma [9].

Pollution from road traffic predominates in urban areas, but rural populations are not safe from exposure to pollution. In 2019, household air pollution from cooking fuels accounted for 697 000 deaths, and ambient air pollution for 394 000 [10]. In LMIC, nearly three billion people rely on biomass for cooking in open stoves inside homes with little or no ventilation [11]. The populations most at risk are women and the young [12]. Biomass combustion produces high levels of pollution with many toxic agents [13], including many fine particles (PM2.5, PM10, etc.) but also carbon monoxide, nitric oxide and volatile organic compounds [14] that are known to be an important risk factor for respiratory allergic diseases including asthma.

People in rural areas of sub-Saharan Africa live not only with outdoor and indoor pollution but also with endemic parasitic diseases. Among the latter, helminths play a prominent role due to their chronicity allied to their ability to regulate the human immune system [15]. Helminth infections are characterized by the induction of Th2-type immune responses resulting in increased interleukin (IL)-4, IL-5, IL-13 and immunoglobulin (Ig) E levels [16]. The same type of response develops in some individuals on contact with allergens, leading to strong inflammatory responses as seen in asthma [17]. However, to ensure their own survival, in their chronic phase helminths establish an immunoregulatory network that limits the Th2-type response of the host. Thus, regulatory T and B cells are activated and high levels of the regulatory cytokine IL-10 are produced. These immunoregulatory elements not only maintain the helminth infection in the host, but also suppress inflammatory responses in infected individuals [18].

It has been hypothesized that increased hygiene, reduced family size, and consequently decreased microbial exposure levels, could explain the increasing global prevalence of asthma [19]. According to this ‘hygiene hypothesis’ reduced exposure to infectious agents may explain the increased incidence of allergic and autoimmune diseases in industrialized countries [20, 21]. The evidence linking helminth infections with allergic diseases is controversial. While several studies have shown inverse associations between helminths and allergy [22–25], a recent review suggested that *Ascaris lumbricoides* infections may increase the risk of bronchial hyperreactivity in participants and of atopy in adults [26]. Notably, helminth elimination as a public health problem is integrated into the WHO roadmap for Neglected Tropical Diseases 2021–2030 [27].

To evaluate the potential contribution of different factors to wheeze in children, we took advantage of the DeWorm3 trial. DeWorm3 is a multi-country (Benin, Malawi and India) community-based cluster-randomized trial which aims to assess the feasibility of interrupting transmission of soil-transmitted helminths (STH) using community mass drug administration with albendazole, compared to standard of care school deworming [28]. The specific objectives of the study described here were, therefore: (i) to determine the proportion of participants with at least one episode of wheezing since birth and in the last 12 months among participants infected or not with STH, (ii) to characterize the exposure of participants to indoor air pollution and (iii) to assess the association between wheezing, exposure to indoor air pollution and STH infection status.

## Methods

### Study population and site description

The study took place in the commune of Comé in southern Benin. The population is rural/semi-urban, with fishing, farming and market trading as principal activities. Exposure to pollutants is essentially from household air pollution. In Benin, the majority of households (98,6%) used solid fuels for cooking (74.6% used wood, 20% charcoal, and 4.1% straw/shrubs/grass) and 1% used gas [29]. Smoking tobacco is not common, with a country-wide prevalence of tobacco smoking in 18–69 years of 5.0% (9.5% in men and 0.5% in women [30].

### Study design and participants

We implemented a cross-sectional survey to assess the presence of wheeze among participants included in the longitudinal monitoring cohort (LMC) of the DeWorm3 study, which took place between August 26th and October 25th, 2020. In the LMC, a total of 6000 individuals were included. We conducted the study described here in a subset of participants aged 6–14 years of age, whose households were located and who were present during the third LMC survey in 2020. A total of 1,130 participants met these criteria. We used data collected at baseline to define STH infection status.

### Data collection

#### Questionnaire on asthma symptoms

Due to considerable concern that allergic conditions were increasing in western and developing countries, a worldwide consortium, the International Study of Asthma and Allergies in Childhood (ISAAC) [31–34] implemented an epidemiological research program which aimed to investigate the prevalence and determinants of asthma, rhinitis and eczema in participants. ISAAC studies use a simple and standardized framework [34, 35] which can be implemented in a wide range of settings with a high standard of replication [36] to know whether participants had experienced wheeze, a symptom that is commonly attributable to asthma, in the preceding 12 months. To help them understand the purpose of the study, we presented to the participants and their parents a 2 min video developed by the ISAAC consortium showing diverse manifestations of asthma-related wheezing [37]. The questionnaire was administered in the local language by a team of five interviewers. Our study questionnaire combined the wheezing module of ISAAC questionnaire, a module on allergy predisposing factors and a module on participants’s exposure to indoor air pollution. Data were collected using Kobocollect software deployed on smartphones and stored in password-protected data sets in secured servers. The wheezing questionnaire database was subsequently merged with the baseline DeWorm3 LMC survey database by the DeWorm3 central data manager. To blind the data, the household, individual, village and cluster identifiers were anonymized and all other identifiers removed.

#### Contact with allergens

These data were collected following the instructions and hypotheses of the ISAAC phase 3 manual using the recommended Environmental Questionnaire [41, 42] that has been adapted to local habits and customs (http://isaac.auckland.ac.nz/).

#### Helminth infection

Data on helminth infection were from the baseline study of the DeWorm3 longitudinal cohort, conducted in April 2018. Participants’ stool samples were screened for the presence of STH using the Kato-Katz technique [38]. The parasite intensity was calculated from a Kato-Katz smear made with 41.7 mg of stool, by multiplying the egg count from the slide by a factor of 24 (24 × 41.7 mg ≈ 1 g) to get the number of eggs per gram of stool (EPG).

#### Treatment information

Between the baseline survey in 2018 and the wheezing questionnaire in 2020, there were in total five rounds of mass drug administration (MDA) for STH using albendazole, with participants treated differently according to their location in intervention (maximum five treatments) or control clusters (maximum two treatments). Assumptions were made regarding treatment status of participants in control clusters, with one school-based treatment (in 2019) for pre-school aged participants (aged 4 years at baseline in 2018) and two school-based treatments (in 2018 and 2019) for school-aged participants.

### Variables

#### Dependent variables

The dependent variables were current wheezing, history of wheezing and severe wheezing. Based on ISAAC studies, current wheezing was defined by at least one episode of wheezing during the last 12 months, and wheezing history was defined by at least one wheezing episode since birth. Severe wheezing was defined as current wheezing with more than four wheezing attacks or more than one night per week of sleep disturbance due to wheezing, or wheezing affecting speech, as assessed through the questionnaire administered [31].

#### Covariates

The covariates of interest were (i) helminth infection treated as a binary variable (yes/no) and intensity of helminth infection (null/light/moderate/heavy) [39] and (ii) variables related to exposure to household air pollution, such as cooking fuels (wood only/charcoal only/gas only/mixed); biomass fuels (wood only/charcoal only/mixed); daily exposure time to cooking fuels (in hours); type of cookstove (opened only/protected only/mixed); cooking place (indoor/outdoor/mixed); fire starters: oil, palm cake, plastic bags; and (iii) proxy of exposure to ambient air pollution, such as distance from home to a paved or unpaved but busy road.

Other covariates were (i) socio-demographic variables including gender; wealth index, a variable corresponding to wealth level quintiles detailed elsewhere [40]; household size (number of individuals in the household); and body mass index (BMI) (kg/m^2^); (ii) exposure to tobacco smoke (yes/no); (iii) allergens variables categorized according to ISAAC environmental questionnaires [41, 42] which included diet (exclusively allergy-protective/exclusively allergy-prone or mixed); contact with animal allergens (dogs, cats, rodents); food products stored in the rooms, where the child lives (yes/no); proximity to volatile toxic products (insecticide (spirals/aerosols etc.), rat poison, oil (yes/no)); type of housing material (natural versus man-made); sleeping material (natural versus man-made); (iv) number of albendazole treatments.

### Statistical analysis

Proportions were computed with 95% confidence intervals and continuous variables were described with means and interquartile ranges (IQR). We used Student's *T* test to compare quantitative variables and Chi^2^ test for qualitative variables. To investigate the association between wheezing and helminth infection status, taking into account air pollution, several regression models were used. We performed a logistic regression with the variable «current wheezing» treated dichotomously (yes/no). A multinomial (polytomous) logistic regression was done with the dependent variable ‘wheezing severity’, a qualitative ordinal variable with three categories (no wheezing/wheezing/severe wheezing). At each of the previous stages, univariate and multivariate analyses were performed. Variables whose *p* value was < 0.2 at the univariate stage were included in the multivariate model. In this last step the significance level was 0.05. The final model with all significant *p* values was obtained by eliminating variables through a step-by-step descending method. Effect sizes are presented with 95% confidence intervals. All analyses were performed with Stata 14 for Windows (Stata Corp., College Station, TX).

### Results

#### Study population

**Fig. 1:**
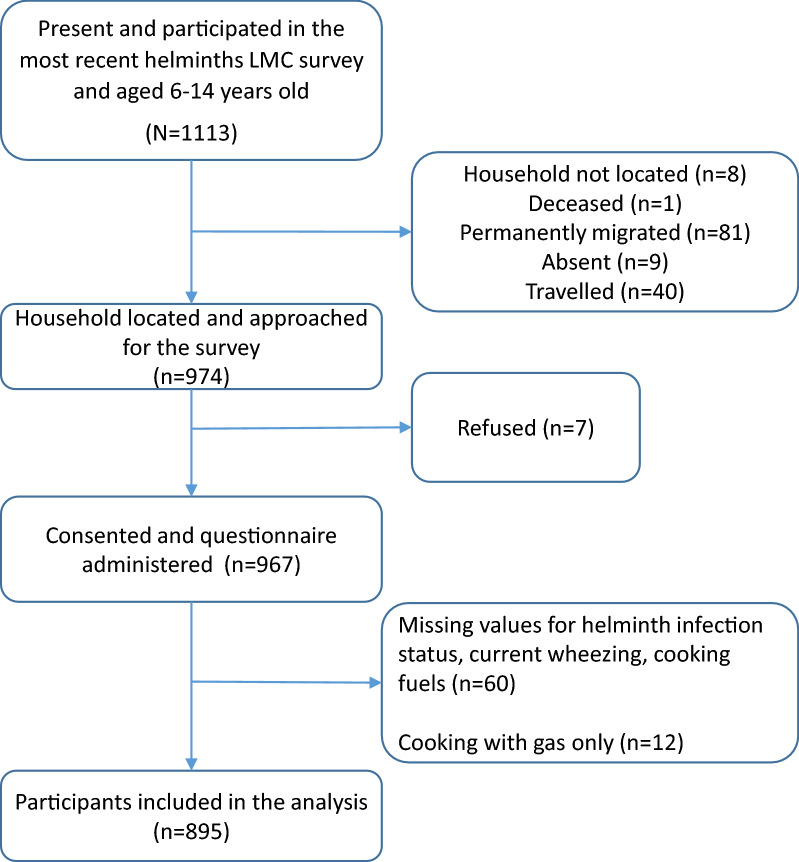
Study flow chart

Among the 1113 participants aged 6–14 years from the DeWorm3 LMC database considered for the wheezing survey, there were 81 participants who had permanently migrated, forty had travelled, eight with households not located and one deceased (Fig. 1). Among the 974 participants we asked for, seven refused to participate, leading to a 99.3% (967/974) participation rate. We excluded from the database observations with missing information for questions concerning current wheezing (*n* = 6), cooking fuels (*n* = 11) and STH infection status (*n* = 55). The descriptive analysis concerned 895 participants.

The median age was 9.2 years (IQR 7.2–11.8), with 46.2% females. Household size ranged from 2 to 20 individuals with a median of 6 (IQR 5–7). Most participants (98.1%) had a normal BMI, i.e., between 18 and 25 kg/m^2^ (Table 1a).

**Table 1:** Description of the study population: socio-demographic characteristics, helminth infection and treatment information, exposure to air pollution, and allergy related variables

Variable	N/Median (IQR)	%/Range
a. Socio-demographic characteristics		
Age (years)	9.2 (7.2–11.8)	6.0–14.9
Gender		
Male	482	53.8
Female	413	46.2
Wealth index		
1st Quintile	149	16.7
2nd Quintile	158	17.6
3rd Quintile	165	18.4
4th Quintile	207	23.1
5th Quintile	216	24.1
Household size (individuals)	6 (5–7)	2–20
Body mass index (kg/m^2^)		
Normal (18–25)	878	98.1
Overweight (25–30)	10	1.1
Obesity (sup 30)	7	0.8

b. Description of the study population: helminth infection and treatment information		
Soil-transmitted helminth (STH) infections		
Ascaris		
No	864	96.5
Yes	31	3.5
Hookworm		
No	878	98.1
Yes	17	1.9
Trichuris		
No	893	99.8
Yes	2	0.2
Any STH		
No	845	94.4
Yes	50	5.6
Intensity of infection with Ascaris—continuous (EPG)	7968 (1680–34296)^a^	12–56412
Intensity of infection with hookworm—continuous (EPG)	84 (48–492)	12–11100
Intensity of infection with Trichuris—continuous (EPG)	10122	120–20124
Intensity of infection with Ascaris		
Null (not infected)	864	96.5
Light (1–4,999 EPG)	13	1.4
Moderate (5,000–49,999 EPG)	17	1.9
Heavy (> 50,000 EPG)	1	0.1
Intensity of infection with Hookworm		
Null (not infected)	878	98.1
Light (1–1,999 EPG)	15	1.7
Moderate (1,000–9,999 EPG)	1	0.1
Heavy (> 10,000 EPG)	1	0.1
Intensity of infection with Trichuris		
Null (not infected)	893	99.8
Light (1–999 EPG)	1	0.1
Moderate (2,000–3,999 EPG)	0	0
Heavy (> 4,000 EPG)	1	0.1
Number of MDA treatments—continuous	2 (0–5)	0–5
Number of MDA treatments (category)		
0–1 treatment	111	12.4
2–3 treatments	363	40.6
4–5 treatments	421	47.0

c. Description of the study population: exposure to air pollution		
Household air pollution		
Exposure to cooking fuels		
All fuels (n = 907)		
LPG only	12	1.3
Wood only	352	38.8
Charcoal only	251	27.7
Mixed fuels	292	32.2
Biomass fuels (n = 895)		
Wood only	352	39.3
Charcoal only	251	28.1
Mixed biomass fuels	292	32.6
Daily exposure time to any cooking fuel (in hours)	0.8 (0.3–5.8)	0.3–8
Daily exposure time to wood burning (in hours)	0.8 (0.5–1.5)	0.3–8
Daily exposure time to charcoal burning (in hours)	0.8 (0.3–1.5)	0.3–6.5
Type of cookstove		
Enclosed only	291	32.5
Open only	410	45.8
Mixed: open or enclosed	194	21.7
Cooking location		
Exclusively indoor (with a roof and walls)	142	15.9
Mixed	344	38.4
Exclusively outdoor	409	45.7
Material used for fire lighting		
Kerosene		
No	670	74.9
Yes	225	25.1
Palm cakes		
No	261	29.1
Yes	634	70.9
Plastic bags		
No	851	95.1
Yes	44	4.6
Second hand smoking		
No	810	90.5
Yes	42	4.7
Unknown/Missing	43	4.8
Ambient air pollution		
Distance from home to asphalt road		
< 50 m	97	10.8
50–200 m	87	9.7
> 200 m	711	79.4
Distance from home to unpaved but busy road		
< 50 m	351	39.2
50–200 m	105	11.7
> 200 m	439	49.0

d. Description of the study population: allergy-related variables		
Variable	Frequency	%
Variables associated with allergies		
Diet		
Exclusively allergy-protective	390	43.6
Exclusively allergy-prone or mixed	501	56.0
Presence of animals (dogs, cats, rodents)		
No	390	43.6
Yes	505	56.4
Foodstuff stored in room (cereals, tubers, nuts)		
No	442	49.4
Yes	453	50.6
Volatiles/toxic substances stored in room (insecticides, oil, gas oil, rat poison…)		
No	523	58.4
Yes	372	41.6
Housing material		
Man-made	600	67.0
Natural	295	33.0
Bedding material		
Man-made	111	12.4
Natural	780	87.1
Unknown/missing	4	0.4

^a^Median (IQR)

^b^Intensity in only positive cases

### Wheeze and STH infection

The prevalence of current wheezing was 4.6% [95%CI 3.4–6.7%], of wheezing history was 5.2% [95%CI 4.0–6.9%] and of severe wheezing was 3.1% [95%CI 2.2–4.5%].

Overall, 50 (5.6%) participants were infected with STH, among whom 31 (3.2%) were infected by *Ascaris* *lumbricoides,* 17 (1.8%) by hookworm and 2 (0.2%) by *Trichuris trichiura*. No STH co-infections were detected. Among those infected with STH, the intensity of *Ascaris* infection was light, moderate or heavy in 13, 17 and 1 participants, respectively, with a median intensity of 7968 eggs per gram (EPG) (IQR 1680–34296, range 12–56412 EPG). Infections with hookworm were mostly of light intensity (15 participants), with 1 moderate and 1 heavy intensity, with a median of 84 EPG, (IQR 48–492, range 12–11100). Concerning *Trichuris*, 1 was of light intensity (120 EPG) and 1 of heavy intensity (20124 EPG) (Table 1b). Due to the very low number of cases, *Trichuris* infections were not taken into account in the analyses.

### Exposure to risk factors

#### Ambient air pollution

The distance from home to the nearest main road was taken as a proxy of exposure to outdoor air pollution. Most (79.4%) participants’ houses were more than 200 m from an asphalt road, while half (49%) lived more than 200 m from an unpaved but busy road (Table 1c).

#### Household air pollution

Cooking was exclusively outdoors for 44% of the participants, 16% indoors and for 38.6% cooking was both indoors and outdoors. Open cookstoves were exclusively used by 45.8% of the participants, while 32.4% were exposed exclusively to protected cookstoves and 13.2% were exposed to both categories. With respect to lighting fires, 70% were exposed to palm cake smoke, 25% to kerosene smoke, and 4.6% to plastic bag smoke (Table 1c). Taking all cookstoves into account, the fuels to which our study population was exposed comprised mainly wood (62.4%) and charcoal (53.7%). Straw/shrubs/grass, gas, wood sawdust and kerosene were less frequent (6.1%, 5.2%, 1.4% and 0.2%, respectively). Three hundred and fifty-two (38.8%) participants were exposed to wood burning only, 251 (27.7%) exposed to charcoal burning only, 12 (1.3%) exposed to gas cooking only and 292 (32.2%) exposed to a mixed combination of cooking fuels. Since burning biomass and its effects on respiratory health is the primary focus of this study, we excluded the 12 participants (1.3%) with only gas-based cooking exposure from the analysis.

#### Contact with allergens

Fifty-six percent of participants (541) were in contact with dogs, cats or rodents. 57% (550) consumed allergy risk foods or a mixed diet in the previous 2 weeks, while 407 participants (42%) consumed allergy protective foods; 323 (33.5%) participants lived in rooms, where no food was stored, while 638 (66.2%) participants lived in rooms, where at least one of two types of food among cereals, tubers and nuts were stored; 409 (42.4%) of the participants lived near volatile toxic products, such as insecticides and petrol; 310 (32.2%) participants lived in a house with a roof, floor or wall made of natural material compared to 654 (67.9%) participants living in a house made of manufactured materials (Table 1d).

#### Mass treatments with albendazole

One hundred and eleven (12.4%) participants received 0 to 1 treatment with albendazole, 363 (40.6%) received 2 to 3 treatments and 421 (47%) received 4 to 5 treatments. Details of albendazole treatment status are available in Table 2.

**Table 2:** Wheezing study participants’ age-group at baseline and treatment arm in DeWorm3

Age-group at Baseline	Intervention arm (community MDA^a^)					Control arm (school MDA)	
	MDA1 (06–2018)	MDA2 (11–2018)	MDA3 (06–2019)	MDA4 (11–2019)	MDA5 (06–2020)	MDA2 (11–2018)	MDA4 (11–2019)
PSAC^b^ (1–4 years)	111/120	114/120	109/120	111/120	111/120	1/106^d^	106/106^e^
SAC^c^ (5–14 years)	322/330	321/330	309/330	309/330	294/330	339/339¤	339/339^e^

^a^MDA: Mass Drug Administration

^b^PSAC: Pre-School Aged Children,

^c^SAC: School-Aged Children

^d^In 2018, there were 106 PSAC aged 4 years in control clusters who were not eligible for treatment. One of them was treated by mistake during community MDA2

^e^Because individual treatment data were not available for MDA in control clusters, we assumed that all children were treated at school

### Factors associated with asthma symptoms

To avoid bias related to memory recall, we focused the analysis on current wheezing and severe wheezing which are related to the 12 previous months.

#### Univariate analysis

Children harboring STH infection (all species) were more likely to suffer current wheezing than those not infected (OR = 5.6 [2.5–12.4]). Among them, infection with *Ascaris* was significantly associated with current wheezing (OR = 7.1 [2.9–17.7]), whereas hookworm infection was not (OR = 1.3 [0.2–10.1]). We found no association between current wheezing and neither intensity of STH infections (OR = 1.0 [0.9–1.0]), nor number of MDA treatments (OR = 0.98 [0.81–1.19]). Being overweight (i.e., BMI 25–30 kg/m^2^) was associated with a higher prevalence of wheezing (OR = 9.5 [2.4–38.1]) compared to normal BMI. However, belonging to the 3rd and 4th quintile of asset index, compared to the 1st quintile representing the poorest, was associated with a significantly lower odds of current wheezing (OR = 0.3 [0.1–0.9] and OR = 0.3 [0.1–0.8], respectively).

Regarding indoor air pollution, participants with longer exposure time to cooking fuels (crude OR = 1.3 [1.0–1.6]), exposed to open stoves (OR = 5.2 [1.8–15.0]) or mixed (open and protected) stoves (OR = 3.9 [1.2–12.8]) compared to protected stoves had a higher risk of current wheezing. Furthermore, using palm cakes to light the fire (OR = 4.1 [1.4–11.7]) was associated with higher odds of a wheezing episode during the 12 past months, whereas using kerosene was associated with lower odds (OR = 0.4 [0.1–0.9]). Exposure to tobacco smoke was not associated with current wheezing.

With respect to outdoor air pollution, participants who lived more than 200 m from an unpaved but busy road were less likely to have wheezing episodes than those closer than 50 m (OR = 0.5 [0.2–0.99]) to the road.

As far as allergy-related variables are concerned, participants in contact with animals (dogs, cats, rodents), living in rooms, where food products (cereals, tubers, nuts) or volatile toxic substances (insecticides, oil, gas oil, rat poison) are stored were more likely to have current wheezing episodes (OR = 3.5 [1.6–7.8]; OR = 2.0 [1.0–3.8] and OR = 3.7 [1.9–7.4], respectively).

#### Multivariate analysis

In multivariate analyses, the association between *Ascaris* infection and current wheezing remained (adjusted odds ratio (aOR) = 4.3 [1.5–12.0]). Other variables that remained in the final model were being overweight (aOR = 9.7 [1.7–55.9]), type of cookstove, open only (aOR = 3.9 [1.3–11.5]) versus improved only, palm cakes for fire lighting (aOR = 3.4 [1.1–9.9]) and contact with animals (dogs, cats, rodents) (aOR = 2.5 [1.1–6.0]), all of them representing risk factors for current wheezing. There was no significant interaction between *Ascaris* infection and either type of cookstoves (Log-likelihood ratio test, p = 0.06) or contact with animals (p = 0.48).

Details of univariate analysis are presented in Additional file 1: Tables S1a, S1b, S1c and S1d and the reduced model after multivariate analysis is presented in Table 3.

**Table 3:** Reduced model of logistic regression showing factors associated with current wheezing

Predictors	Current wheezing					
	Univariate analysis			Multivariate analysis		
	Crude Odds Ratio	95% CI	p value	Adjusted^a^ Odds Ratio	95% CI	*p* value
Socio-demographics						
Body mass index (kg/m^2^)						
Normal (18–25)	Ref.			Ref.		
Overweight (25–30)	9.5	2.4–38.1	0.002	9.7	1.7–55.9	0.011
Obesity (> 30)	1			1	-	-
STH infection						
Ascaris						
No	Ref.			Ref.		
Yes	7.1	2.9–17.7	0.001	4.3	1.5–12.0	0.006
Household air pollution						
Exposure to cooking fuels						
Type of cookstove			0.001			
Enclosed only	Ref.			Ref.		
Open only	5.1	1.7–14.6	0.003	3.9	1.3–11.5	0.014
Mixed: open or enclosed	3.9	1.2–12.6	0.02	2.9	0.9–9.6	0.088
Material for fire lighting						
Palm cakes						
No	Ref.			Ref.		
Yes	4.0	1.4–11.3	0.009	3.4	1.1–9.9	0.028
Variables associated with allergies						
Presence of animals (dogs, cats, rodents)						
No	Ref.			Ref.		
Yes	3.9	1.7–9.0	0.001	2.5	1.1–6.0	0.034

^a^Adjusted on sex, wealth index, type of cooking fuel, exposure time to combustion smoke, cooking location, fire lighting with kerosen, outdoor air pollution (distance from house to road traffic), diet, stoarge of foodstuff (cereals, tubers, nuts) or volatiles/toxic substances (insecticides, oil, gas oil, rat poison) in room, nature of housing materials,

### Factors associated with severe wheezing

We investigated the factors potentially associated with severe wheezing using multinomial (polytomous) logistic regression. Multivariate analysis revealed the same trend as for current wheezing in terms of associated factors with the exception of palm cakes for fire lighting that was not associated with severe wheezing. Indeed, participants with *Ascaris* infection had higher odds of severe wheezing (aOR = 9.2 [3.1–27.8]), as did those exposed to open cookstoves only versus improved only (aOR = 3.9 [1.1–13.7]) and those who were overweight (aOR = 10.3 [1.8–60.0]). The reduced model after multivariate analysis is presented in Table 4.

**Table 4:** Factors associated with severe wheezing using multinomial logistic regression

Predictors		Multivariate analysis		
		Adjusted Odds Ratio^a^	95%CI	*p* value
No wheezing		Ref.		
Severe wheezing	Infection with Ascaris			
	No	Ref.		
	Yes	**9.2**	**3.1–27.8**	** < 0.001**
	Type of cookstove			
	Enclosed only	Ref.		
	Open only	**3.9**	**1.1–13.7**	**0.03**
	Mixed: open or enclosed	1.7	0.4–7.8	0.47
	Body Mass Index (kg/m^2^)			
	Normal (18–25)	Ref.		
	Overweight (25–30)	**10.3**	**1.8–60.0**	**0.01**
	Obesity (> 30)	1.8 × 10^–6^	–	0.99
	Presence of animals (dogs, cats, rodents)			
	No	Ref.		
	Yes	2.3	0.8–6.4	0.11

The bold values in table 4 represent the significant values

^a^Adjusted on sex, type of cooking fuel, exposure time to combustion smoke, cooking place, fire lighting with kerosen or palm cakes, diet, storage of foodstuff (cereals, tubers, nuts) or volatiles/toxic substances (insecticides, oil, gas oil, rat poison) in room

## Discussion

In the study described here, we investigated the factors associated with asthma symptoms in children living in a rural/semi-urban setting of southern Benin, focusing on soil-transmitted helminth (STH) infections as well as exposures to environmental factors, such as air pollution and allergens. Our study is thus distinctive—we know of no other published study to have assessed such a combination of factors in the context of asthma symptoms in a sub-Saharan African setting. The study leveraged the DeWorm3 trial in which the study subjects were participants. DeWorm3 is designed to assess STH transmission interruption through community mass deworming [28, 43].

In our study population the proportion with a history of wheeze was 5.2% and with current wheezing of 4.6%. Similar prevalences have previously been reported in children living in northern Benin [44]. In the Phase 3 ISAAC study, the prevalence of current wheezing in the African settings assessed was 10% and 14% in 6–7 year and 13–14 year age groups, respectively [31]. The Phase 3 ISAAC Centers assessed urban residents as opposed to the predominantly rural residents of our study site, a difference that, we speculate, possibly explains the difference in proportions of those found with wheezing. The prevalence of severe wheezing was 3.1% in our population, and the ratio between current wheezing and severe asthma is, therefore, consistent with the results of the third ISAAC study [31], showing that the proportion of those with current wheeze presenting clinical signs of severe asthma was higher in Africa than in other regions. The reasons could be poorer asthma care in low income countries together with lower awareness of wheeze being a symptom of asthma. However, differences of exposure to both air pollutants and infectious agents may also contribute to the greater severity observed in LMIC [31]. This difference underlines the importance of performing more studies in Benin and more generally in LMIC using the ISAAC questionnaire and tools.

The marked positive association we found between symptoms of wheeze and *A. lumbricoides* infection, with no association—either positive or negative—found for hookworm, are observations that are consistent with the findings of other studies conducted in sub-Saharan African settings [45, 46], although inverse associations between hookworm and allergic disease have nevertheless been reported in Uganda, for example, [47, 48]. Since a seminal study in Gabon on the topic [49], with the principal findings subsequently reproduced elsewhere [50], helminths in general have been thought to offer protection from allergic diseases. This is related to the fact that, to ensure their survival, these parasitic worms modulate the human immune system in the chronic phase of their infection through induction of IL-10-driven immunoregulatory responses that act, in a so-called ‘bystander’ way, to suppress allergic inflammation [15–17]. Thus, chronic helminth infections may protect from allergic diseases including asthma [51–54]. In such a scenario our results with respect to wheeze and *A. lumbricoides* could be seen as counter-intuitive, but they are, nevertheless, entirely in accordance with the conclusions drawn by reviews of recent studies on the topic of associations between STH and symptoms of asthma, including those of a systematic nature with meta-analyses [26, 55, 56].

Mechanistically, a precise immunological explanation for the association between wheeze and *A. lumbricoides* remains obscure, but a relationship with the inflammatory response to the pulmonary passage of larvae of *A. lumbricoides* seems the most plausible. It is known, for example, that *Ascaris* larval migration through airways may be associated with respiratory symptoms, such as wheezing, dyspnoea and bronchospasm [57, 58]. Separately, *Ascaris* is also associated with increased Th2-type and IgE responses to cross-reactive house dust mite(HDM)-specific allergens [59, 60], responses that themselves represent a positive risk factor for asthma and asthma severity [61–63]. That being said, it should be borne in mind that our study population was participating in the DeWorm3 trial involving repeated treatment with the anthelmintic albendazole. Purely from the perspective of STH infections, then, any treatment-mediated decline in the prevalence of *A. lumbricoides* would be expected to be accompanied by a parallel decrease in the prevalence of wheezing. Although, as mentioned earlier, the prevalence of wheezing we observed here is similar to that reported in northern Benin, we do not know if it was actually higher in our study participants prior to implementation of anti-STH treatment in the study site. What remains also unclear is the speed with which such asthma-related symptoms would anyway be expected to resolve following clearance of worms. That wheezing was not associated with the number of treatments children received in our study over the 2 years prior to administering the ISAAC questionnaire could be interpreted as evidence that symptoms do not resolve rapidly. In the same context, intensive anti-STH treatment given every 3 months over a 2 year period in a rural area of Indonesia with a very high worm burden did not affect reported allergy symptoms [64], although it should be noted that the prevalence of STH infections remained relatively high in that study’s population.

We found a strong positive association—independent of STH infection—between wheeze and either exclusive use of open cookstoves, or use of palm cakes for lighting fires, findings that are consistent with earlier studies in Latin America [65, 66]. Open cookstoves use principally biomass as fuel, the incomplete combustion of which generates high levels of pollutants that are harmful to the lung [14]. From a mechanistic perspective, air pollutants cause oxidative stress to the airways, leading to inflammation, remodeling, and increased risk of sensitization [67]. The degree of openness of the cookstove is positively associated with the level of personal exposure to fine particulate matter [68]. Thus, improved cookstoves offer better combustion of the fuel by providing an insulated combustion chamber around and above the fire, enhancing the temperature of the fire and resulting in decreased CO and PM levels compared to open cookstoves [69–71], associated with a reduction in wheezing symptoms both in mothers [72] and children [73]. In contrast to earlier studies [74], here we found no association between wheezing and indoor exposure to tobacco smoke, probably due to the comparatively low prevalence of tobacco smoking in our setting. We also found that being overweight was also positively and independently associated with current wheezing and severe wheezing episodes. Similar findings in children under five have been reported [75]. It is known that fat accumulates inside the airways of overweight individuals, altering their normal structure and leading to inflammation. There is also evidence of positive correlations between BMI, adipose tissue area on airway walls, airway wall thickness and the number of inflammatory cells [76].

There are a number of limitations of our study. One is that both exposure (air pollution, diet, allergens etc.) and wheeze were assessed based not on objective measures but on a questionnaire that could generate memory bias. The ISAAC questionnaire we used is nevertheless a highly standardized tool that has been used internationally for decades, facts that more than 500 peer reviewed publications attest to. Another limitation concerns the trial’s blinding context that prevented use of participants’ post-intervention helminth infection status. The true helminth prevalence at the time of administration of the ISAAC questionnaire would, logically, be expected to have been lower than the baseline level. In addition, the relatively low prevalence of the outcome measure, wheezing, may also have limited the study’s power to detect potentially meaningful associations, while the repeated treatment of children with albendazole may have modified some of the associations detected by decreasing the intensity of helminth infections.

To our knowledge, this study is the first to investigate risk factors for wheeze taking into account the potential role of both helminths and household air pollution (HAP) in the same model. HAP induces airway inflammation that elicits asthma symptoms, a process that our findings are consistent with. In the context of the epidemiological transition phenomenon ongoing in LMIC-like Benin, our work clearly highlights the importance, in future studies, of considering helminth infection characteristics (species, prevalence, intensity), combined with assessments of individuals’ exposure to HAP as well as their biometrics, to better determine potential risk factors for wheeze in childhood and adolescence.

## Conclusions

Many LMIC countries are facing an epidemiological transition with an increase in the prevalence of non-communicable diseases including asthma. Understanding the risk factors for asthma in the tropics is important for tailoring the public health response. Such an understanding necessitates taking into account environmental factors, including exposure to air pollution as well as to potential allergens, and helminth infections that may differentially affect inflammatory responses through allergy-inducing and/or immunomodulatory effects. With respect to preventing wheezing, our study highlights potentially modifiable factors of public health interest that could include promoting the use of improved cookstoves, preventing overweight in children, and preventing contact with domestic animals. Perhaps of most relevance with regard to ongoing debates concerning the benefits of mass deworming programmes, our results clearly demonstrate added value in terms of reducing the prevalence of *Ascaris* infections that can precipitate wheezing.

## Supplementary Information

**Additional file 1: Table S1a**: Socio demographic factors univariately associated with current wheezing using logistic regression. **Table S1b**: Helminth infection and treatment-related factors associated with current wheezing using univariate logistic regression. **Table S1c**: Air pollution-related factors associated with current wheezing using univariate logistic regression. **Table S1d**: Allergy-related factors associated with current wheezing using univariate logistic regression.

## Data Availability

Under agreement with the IRBs of the Deworm3 study, data must be blinded until the study concludes. Therefore, to avoid breaching the agreement with the ethical approval bodies, data cannot be shared publicly, because the study remains blinded to outcome data. Data are available from the DeWorm3 Institutional Data Access Committee (contact via dw3data@uw.edu) for researchers who meet the criteria for access to these data. Data exclusively related to the wheezing study can be made available upon request to the corresponding author.
