# Supplementary material for: Multiple overlapping risk factors for childhood wheeze among children in Benin
Source: Eur J Med Res. 2022 Dec 26;27:304. doi: 10.1186/s40001-022-00919-1 (PMC9791764; doi:10.1186/s40001-022-00919-1)
Supplement: Supplementary file 1 — Additional file 1: Table S1a: Socio demographic factors univariately associated with current wheezing using logistic regression. Table S1b: Helminth infection and treatment-related factors associated with current wheezing using univariate logistic regression. Table S1c: Air pollution-related factors associated with current wheezing using univariate logistic regression. Table S1d: Allergy-related factors associated with current wheezing using univariate logistic regression. [file 40001_2022_919_MOESM1_ESM.docx]

| **SUPPLEMENTS** |
| --- |

Table S1a : Socio demographic factors univariately associated with current wheezing using logistic regression

|  | **Univariate Logistic Regression** | | |
| --- | --- | --- | --- |
| **Predictors** | **Crude Odds Ratio** | **95% CI** | **p value** |
| **Socio-Demographics** |  |  |  |
| Age (years) | 1.1 | 0.9-1.2 | 0.24 |
| Sex |  |  |  |
| Male | Ref |  |  |
| Female | 0.7 | 0.3-1.3 | 0.21 |
| Wealth index |  |  | 0.10 |
| 1st Quintile (poorest) | Ref |  |  |
| 2nd Quintile | 0.5 | 0.2-1.2 | 0.13 |
| 3rd Quintile | 0.3 | 0.1-0.9 | 0.04 |
| 4th Quintile | 0.3 | 0.1-0.8 | 0.02 |
| 5th Quintile (richest) | 0.5 | 0.2-1.2 | 0.12 |
| Household size | 1.0 | 0.9-1.1 | 0.71 |
| Body Mass Index (kg/m²) |  |  |  |
| Normal (18-25) | Ref |  |  |
| Overweight (25-30) | 9.5 | 2.4-38.1 | 0.002 |
| Obesity (> 30) | 1 |  |  |

Table S1b : Helminth infection and treatment-related factors associated with current wheezing using univariate logistic regression

|  | **Univariate Logistic Regression** | | |
| --- | --- | --- | --- |
| **Predictors** | **Crude Odds Ratio** | **95% CI** | **p value** |
| **STH infection** |  |  |  |
| Ascaris |  |  |  |
| No | Ref |  |  |
| Yes | 7.1 | 2.9-17.7 | 0.001 |
| Hookworm |  |  |  |
| No | Ref |  |  |
| Yes | 1.3 | 0.2-10.1 | 0.80 |
| Trichuris |  |  |  |
| No | Ref |  |  |
| Yes | 21.3 | 1.3-347.1 | 0.03 |
| Any STH |  |  |  |
| No | Ref |  |  |
| Yes | 5.6 | 2.5-12.4 | 0.001 |
|  |  |  |  |
| Intensity of Ascaris infection-continuous (EPG) ** | 1.0 | 0.9-1.0 | 0.09 |
| Intensity of Hookworm infection-continuous (EPG) ** | 1.0 | 0.9-1.0 | 0.79 |
| Intensity of Trichuris infection-continuous (EPG) ** | 1.0 | 0.9-1.0 | 0.87 |
| Intensity of infection with Ascaris-categorized |  |  |  |
| Null | Ref |  |  |
| Light (1-4,999 EPG) | 10.8 | 3.2-37.0 | 0.001 |
| Moderate (5,000–49,999 EPG) | 3.2 | 0.7-14.8 | 0.13 |
| Heavy (*>*50,000 EPG) | 1 | - | - |
| Intensity of infection with Hookworm-categorized |  |  |  |
| Null | Ref |  |  |
| Light (1-1,999 EPG) | 1.5 | 0.19-11.7 | 0.70 |
| Moderate (1,000–9,999 EPG) | 1 | - | - |
| Heavy (*>*10,000 EPG) | 1 | - | - |
|  |  |  |  |
| Any STH |  |  |  |
| No | Ref |  |  |
| Yes | 5.6 | 2.5-12.4 | 0.001 |
| Number of MDA treatments (continuous) | 0.98 | 0.81-1.19 | 0.86 |
| Number of MDA treatments (category) |  |  |  |
| 0-1 treatment | Ref |  |  |
| 2-3 treatments | 2.2 | 0.65-7.55 | 0.21 |
| 4-5 treatments | 1.5 | 0.43-5.26 | 0.51 |

Table S1c : Air pollution-related factors associated with current wheezing using univariate logistic regression.

|  | **Univariate Logistic Regression** | | |
| --- | --- | --- | --- |
| **Predictors** | **Crude Odds Ratio** | **95% CI** | **p value** |
| **Household air pollution** |  |  |  |
| **Exposure to cooking fuels** |  |  |  |
| Biomass fuels |  |  |  |
| Mixed cooking fuels | Ref |  |  |
| Wood only | 1.4 | 0.7-2.7 | 0.35 |
| Charcoal only | 0.3 | 0.1-0.9 | 0.048 |
| Exposure time to cooking fuels (hours) | 1.4 | 1.1-1.7 | 0.01 |
| Type of cookstove |  |  | 0.001 |
| Enclosed only | Ref |  |  |
| Open only | 5.1 | 1.7-14.6 | 0.003 |
| Mixed: open or enclosed | 3.9 | 1.2-12.6 | 0.02 |
| Cooking location |  |  | 0.47 |
| Exclusively indoor (with a roof and walls) | Ref |  |  |
| Mixed | 0.6 | 0.2-1.4 | 0.22 |
| Exclusively outdoor | 0.7 | 0.3-1.6 | 0.43 |
| Material for fire lighting |  |  |  |
| Kerosene |  |  |  |
| No | Ref |  |  |
| Yes | 0.4 | 0.1-1.03 | 0.06 |
| Palm cakes |  |  |  |
| No | Ref |  |  |
| Yes | 4.0 | 1.4-11.3 | 0.009 |
| Plastic bags |  |  |  |
| No | Ref |  |  |
| Yes | 1.0 | 0.2-4.2 | 0.99 |
| **Second hand tobacco smoking** |  |  |  |
| No | Ref |  |  |
| Yes | 0.5 | 0.1-3.6 | 0.48 |
| **Ambient air pollution** |  |  |  |
| Distance from home to asphalt road |  |  | 0.05 |
| <50m | Ref |  |  |
| 50-200m | 3.4 | 0.3-33.6 | 0.29 |
| >200m | 5.3 | 0.7-38.8 | 0.10 |
| Distance from home to unpaved but busy road |  |  | 0.08 |
| <50m | Ref |  |  |
| 50-200m | 1.1 | 0.5-2.7 | 0.78 |
| >200m | 0.5 | 0.2-0.97 | 0.04 |

Table S1d : Allergy-related factors associated with current wheezing using univariate logistic regression.

|  | **Univariate Logistic Regression** | | |
| --- | --- | --- | --- |
| **Predictors** | **Crude Odds Ratio** | **95% CI** | **p value** |
| **Variables associated with allergies** |  |  |  |
| Diet |  |  |  |
| Exclusively allergy-protective | Ref |  |  |
| Exclusively allergy-prone or mixed | 0.6 | 0.3-1.1 | 0.11 |
| Presence of animals (dogs, cats, rodents) |  |  |  |
| No | Ref |  |  |
| Yes | 3.9 | 1.7-9.0 | 0.001 |
| Foodstuff stored in room (cereals, tubers, nuts) |  |  |  |
| No | Ref |  |  |
| Yes | 1.9 | 1.0-3.7 | 0.049 |
| Volatiles/toxic substances stored in room (insecticides, oil, gas oil, rat poison…) |  |  |  |
| No | Ref |  |  |
| Yes | 3.6 | 1.8-7.1 | 0.001 |
| Housing materials |  |  |  |
| Man-made | Ref |  |  |
| Natural | 1.5 | 0.8-2.8 | 0.24 |
| Bedding materials |  |  |  |
| Man-made | Ref |  |  |
| Natural | 1.2 | 0.5-3.0 | 0.67 |
